# Preemptive Living-Related Kidney Transplantation Is a Cost-Saving Strategy Compared With Post-dialysis Kidney Transplantation in Thailand

**DOI:** 10.3389/fmed.2022.869535

**Published:** 2022-07-08

**Authors:** Atthaphong Phongphithakchai, Pochamana Phisalprapa, Chayanis Kositamongkol, Nalinee Premasathian, Nuttasith Larpparisuth, Peenida Skulratanasak, Attapong Vongwiwatana

**Affiliations:** ^1^Division of Nephrology, Department of Medicine, Faculty of Medicine Siriraj Hospital, Mahidol University, Bangkok, Thailand; ^2^Division of Nephrology, Department of Internal Medicine, Faculty of Medicine, Prince of Songkla University, Hat Yai, Thailand; ^3^Division of Ambulatory Medicine, Department of Medicine, Faculty of Medicine Siriraj Hospital, Mahidol University, Bangkok, Thailand

**Keywords:** cost-effectiveness, cost-savings, cost-utility, preemptive kidney transplantation, Thailand

## Abstract

**Background:**

Compared with other kidney replacement therapies, preemptive kidney transplantation (KT) provides better clinical outcomes, reduces mortality, and improves the quality of life of patients with end-stage kidney disease (ESKD). However, evidence related to the cost-effectiveness of preemptive living-related KT (LRKT) is limited, especially in low- and middle-income countries, such as Thailand. This study compared the cost-effectiveness of LRKT with those of non-preemptive KT strategies.

**Methods:**

Cost and clinical data were obtained from adult patients who underwent KT at Siriraj Hospital, Mahidol University, Thailand. A decision tree and Markov model were used to evaluate and compare the lifetime costs and health-related outcomes of LRKT with those of 2 KT strategies: non-preemptive LRKT and non-preemptive deceased donor KT (DDKT). The model’s input parameters were sourced from the hospital’s database and a systematic review. The primary outcome was incremental cost-effectiveness ratios (ICERs). Costs are reported in 2020 United States dollars (USD). One-way and probabilistic sensitivity analyses were performed.

**Results:**

Of 140 enrolled KT patients, 40 were preemptive LRKT recipients, 50 were non-preemptive LRKT recipients, and the rest were DDKT recipients. There were no significant differences in the baseline demographic data, complications, or rejection rates of the three groups of patients. The average costs per life year gained were $10,647 (preemptive LRKT), $11,708 (non-preemptive LRKT), and $11,486 (DDKT). The QALY gained of the preemptive option was 0.47 compared with the non-preemptive strategies. Preemptive LRKT was the best-buy strategy. The sensitivity analyses indicated that the model was robust. Within all varied ranges of parameters, preemptive LRKT remained cost-saving. The probability of preemptive LRKT being cost-saving was 79.4%. Compared with non-preemptive DDKT, non-preemptive LRKT was not cost-effective at the current Thai willingness-to-pay threshold of $5113/QALY gained.

**Conclusions:**

Preemptive LRKT is a cost-saving strategy compared with non-preemptive KT strategies. Our findings should be considered during evidence-based policy development to promote preemptive LRKT among adults with ESKD in Thailand.

## Introduction

End-stage kidney disease (ESKD) is a major health problem in Thailand. Its incidence continues to increase according to Thai Renal Replacement Therapy Registry data (1). From 2007 to 2019, there was nearly a fourfold rise in the number of kidney replacement therapy patients (40,845 to 151,343 cases) (2).

Kidney transplantation (KT) decreases the mortality rate, improves the quality of life, and reduces the costs of treatment of patients with ESKD compared with other kidney replacement therapies (3–5). The organs for KT comes from two sources: decreased donors and living donors. Additionally, KT can be categorized according to dialysis initiation time into two subgroups. (1) preemptive KT, which defined as transplantation performed before initiation of maintenance dialysis and (2) non-preemptive kidney transplantation defined as transplantation performed after initiation of maintenance dialysis. In Thailand, currently, there are three types of available KT consisted of non-preemptive deceased-donor KT (DDKT), non-preemptive living-related KT (LRKT), and preemptive LRKT (6). Thailand has three public health insurance schemes: the Civil Servant Medical Benefit Scheme, the Social Security System, and the Universal Coverage Scheme. These schemes cover the costs of all KT strategies. KTs are available in 191 public hospitals throughout Thailand (6), which is sufficient for Thai patients with ESKD who need to undergo KT. In 2019, Thailand had 729 new cases of KT (557 DDKT and 172 LRKT), with a cumulative number of 6212 cases. The local 10-year graft survival rates and 10-year survival rates were 83.3 and 87.9% for non-preemptive DDKT versus 93 and 87.9% for LRKT, respectively. The waiting time for KT was approximately 5 years for non-preemptive DDKT versus 2.3 years for LRKT (6). To shorten the waiting period for KT, preemptive LRKT is a good treatment option. There is evidence that non-preemptive LRKT is more beneficial than non-preemptive DDKT. Nevertheless, in the past decade, the use of non-preemptive LRKT was less than that for non-preemptive DDKT (19 vs. 81%) (6).

In addition, previous studies reported that patients receiving preemptive LRKT had better allograft survival, better post-transplant kidney function, and lower overall treatment costs than patients with non-preemptive KT (7–9). Another crucial benefit of preemptive LRKT was to avoid complications associated with dialysis, such as severe infection and mortality during the first 90 days of dialysis (10, 11). Economic evaluations of KT have been conducted and used for policymaking in many developed countries (12–14). However, there have been no studies on the cost-utility of preemptive LRKT in Thailand and other low- and middle-income countries. Our study aimed to compare the cost-utility of preemptive LRKT with those of non-preemptive LRKT and DDKT in Thailand.

## Materials and Methods

### Study Design

A cost-utility analysis was performed using published evidence and primary data from a newly conducted retrospective cohort study at the largest university hospital in Thailand (Siriraj Hospital, Mahidol University) (15). The study enrolled adults aged at least 18 years who underwent the KT procedure between January 2009 and December 2019. The exclusion criteria were multiple organ transplantation and a lack of available medical record data of more than 80%. Economic evaluations complied with the Consolidated Health Economic Evaluation Reporting Standards (CHEERS) statement (Appendix 1). Costs and transitional probabilities were derived from the newly conducted cohort. Other input parameters (age-specific mortality rate, standardized mortality ratios, and utilities) were based on published literature. The details are below.

### Description of the Economic Model and Economic Evaluations

The cost-utility analysis approach was used for the economic evaluations to compare the costs and outcomes (in quality-adjusted life years [QALY]) of preemptive LRKT, non-preemptive LRKT, and non-preemptive DDKT in adult patients with ESKD, aged 50 years and over. For non-preemptive KT strategies, we assumed that the maximum age when KT could be performed was 65 years. The scope of this study was based on current policies under which financial support is provided to offset the expenses associated with preemptive and non-preemptive KTs performed by any Thai government-operated healthcare provider. Nevertheless, the preemptive strategy was not regularly performed since data on its costs, effectiveness, and health economics in Thailand were limited. An economic model, analysis, and outcome validation were conducted by a research team including nephrologists, epidemiologist, and health economist.

**FIGURE 1:**
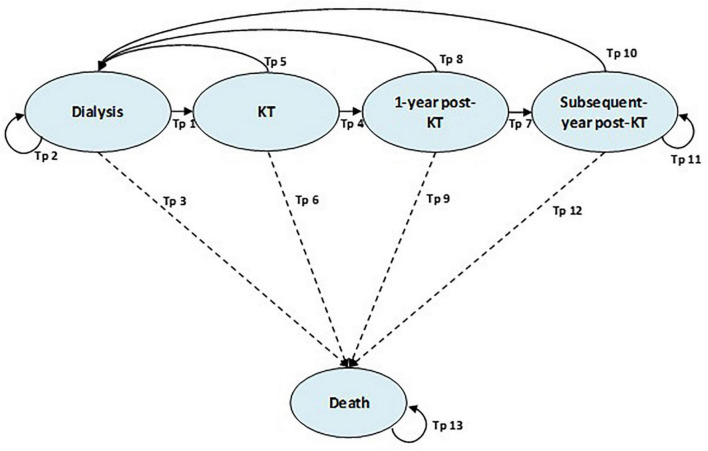
Markov model. KT, kidney transplantation; Tp, transitional probability.

A decision tree and Markov model were developed to compare three KT options: (1) preemptive LRKT, (2) non-preemptive LRKT, and (3) non-preemptive DDKT. Patients were divided into three pathways in a decision tree based on the KT strategy used at their first admission. The Markov model was applied to capture the lifetime costs and outcomes after we classified the patients with ESKD into the three KT strategies using the decision tree. The Markov model consisted of five health states representing the natural course of patients with ESKD who would undergo KT. They were (1) dialysis, (2) KT, (3) 1-year post-KT, (4) subsequent-years post-KT, and (5) deaths (Figure 1). There were 13 transitional probabilities (Tp) as dialysis to KT (Tp1), dialysis to dialysis (Tp2), dialysis to death (Tp3), KT to 1-year post-KT (Tp4), KT to dialysis (Tp5), KT to death (Tp6), 1-year post-KT to subsequent-year post-KT (Tp7), 1-year post-KT to dialysis (Tp8), 1-year post-KT to death (Tp9), subsequent-year post-KT to dialysis (Tp10), subsequent-year post-KT to subsequent-year post-KT (Tp11), subsequent-year post-KT to death (Tp12), and death to death (Tp13). This model was based on an article by Bayani et al. (16), which presented the first model-based economic evaluation utilizing real-world data in the context of the Philippines. The arrows between the health states in Figure 1 represent the ability to transition between the health states in each cycle of the model. The 1-year cycle length and a lifetime time horizon were used in the Markov model. We compared the cost-effectiveness of preemptive LRKT versus non-preemptive DDKT as the base-case analysis. The other two scenario analyses were comparisons of (1) preemptive LRKT versus non-preemptive LRKT and (2) non-preemptive LRKT versus non-preemptive DDKT. All patients who received preemptive LRKT entered the model at KT health state. Then, they would return to dialysis health state only if they failed KT. Those underwent non-preemptive KT strategies entered the model at dialysis health state. The transitional probabilities of shifting from dialysis to KT determined when they would undergo KT.

Our results are presented as incremental cost-effectiveness ratios (ICERs) in 2020 United States dollars (USD) per quality-adjusted life year (QALY) gained. A willingness-to-pay (WTP) threshold of $5113/QALY was used to interpret the cost-effectiveness of an intervention (17). Additionally, an intervention that provided more outcomes but was less costly than another was considered a cost-saving intervention. Costs and outcomes were discounted at an annual rate of 3%, as suggested by the Thai Health Technology Assessment (HTA) Guideline (17).

We performed a one-way sensitivity analysis by changing the value of each input parameter within the 95% confidence interval. Where 95% confidence interval was not available, range ± 15% from base-case value was applied for sensitivity analysis (Table 1). Discount rates ranged from 0 to 6% per year. The results of the one-way sensitivity analysis are presented as tornado diagrams. Moreover, a multivariate probabilistic sensitivity analysis (PSA) was performed using 1,000 Monte Carlo simulations using Microsoft Excel 2019 (Microsoft Corp., Redmond, WA, United States) (18), in which transition probabilities, costs, and utilities were varied. Transitional probabilities and utilities were assigned a beta distribution, while cost data were assigned a gamma distribution. The results of the PSA are presented as a cost-effectiveness plane and cost-effectiveness acceptability curves (CEAC) to show the probability of treatment with preemptive LRKT being cost-effective compared with non-preemptive KT strategies.

**TABLE 1:** Input parameters of model.

Input parameters	Base case	SE or range	Distribution	Source
**Annual discounted rate**	0.03	0.00–0.06		
Age of stop at kidney transplantation (yr)	65	–		
Transition probabilities				
- Probabilities of shifting from dialysis to kidney transplantation in LRKT	0.36	0.084	Beta	Database
- Probabilities of shifting from dialysis to kidney transplantation in DDKT	0.18	0.041	Beta	Database
- Probabilities of graft loss in 1 yr	0.04	0.004	Beta	Database
- Probabilities of graft loss in subsequent years	0.01	0.001	Beta	Database
**Mortality**				
- Standardized mortality ratio of patient with ESKD vs general population				(17)
50–59 years	8.6	0.3061		
60–69 years	4.6	0.2041		
70–79 years	1.9	0.1531		
≥ 80 years	7.8	0.1531		
- Pooled risk mortality ratio of patients with ESKD vs KT	2.19	0.352		(18,19)
**Costs**				
**Preemptive LRKT**				Database
- Total cost of dialysis (USD/yr)	13 734	2060	Gamma	
- Total cost of KT (USD/visit)	7816	507	Gamma	
- Total cost of 1-year post KT (USD/yr)	17 592	1139	Gamma	
- Total cost of subsequent year (USD/yr)	9886	584	Gamma	
- Total cost of waiting time (USD)	6879	917	Gamma	
**Non-preemptive LRKT**				Database
- Total cost of dialysis (USD/yr)	13 734	2060	Gamma	
- Total cost of KT (USD/visit)	9104	1070	Gamma	
- Total cost of 1-year post KT (USD/yr)	15 453	1067	Gamma	
- Total cost of subsequent year (USD/yr)	11 709	825	Gamma	
**Non-preemptive DDKT**				Database
- Total cost of dialysis (USD/yr)	13 734	2060	Gamma	
- Total cost of KT (USD/visit)	13 908	1545	Gamma	
- Total cost of 1-year post KT (USD/yr)	19 520	1361	Gamma	
- Total cost of subsequent year (USD/yr)	10 478	440	Gamma	
Dialysis (USD/yr)	12 193	4236	Gamma	(22)
Complication from dialysis (USD/mo)	481	481	Gamma	
Direct non-medical cost (USD/yr)	1059	257	Gamma	
**Utilities**				
Dialysis	0.68	0.1	Beta	(13,16,22)
KT	0.781	0.117	Beta	
1-year post KT	0.889	0.133	Beta	
Subsequent year post KT	0.889	0.133	Beta	

*ESKD, end-stage kidney disease; DDKT, deceased-donor kidney transplantation; KT, kidney transplantation; LRKT, living-related kidney transplantation; mo, month; yr, year.*

### Model Input Parameters

The input parameters consisted of transitional probabilities, age-specific mortality rate, costs, and utilities. The transitional probabilities, direct medical costs, and utilities were obtained from our cohort and published studies (19–22). The input parameters and their sources are detailed in Table 1.

The transitional probabilities of the KT state shifting to the dialysis state were calculated from our 10-year cohort. The age-specific mortality rate of the Thai population was derived from the Life Table of the World Health Organization (19). Due to a lack of local data, the hazard ratios of mortality of KT patients and ESKD patients were based on data from a study by Choi et al. (20). Their large cohort study evaluated the standardized mortality of ESKD patients in a Korean setting. Their findings were used to calculate the mortality rates of our ESKD and KT patients (Table 1).

### Utility Data

The utility of each of the 4 health states (dialysis, KT, 1-year post-KT, and subsequent-year post-KT) was calculated based on published literature (16, 23, 24). We estimated the utility of all patients in the dialysis state based on the Thailand public health survey conducted by Teerawattananon et al. (23). We also estimated the utility of KT based on previous literature (16, 24). Bavanandan et al. (24) measured utilities using the EQ-5D-3L questionnaire (25) in pre- and post-KT Malaysian populations; a total of 118 adult patients were assessed. The results did not show significantly increased utilities due to the high baseline utility of 0.91 pre-transplantation. The authors explained that the high utility might have been due to excluding patients over 60 years of age and with major comorbidities from the DDKT waiting list. Bayani et al. (16) studied the utility of patients with ESKD using the EQ-5D-5L tool (26) among 45 KT patients in the Philippines. KT was reported to have a higher utility value (0.91) than hemodialysis (0.68) and peritoneal dialysis (0.78). Our utility estimates were based on these studies, and we assumed that 1-year post-KT patients had the same utility as patients in the subsequent KT state.

### Cost Data

The societal perspective was adopted in this study. However, indirect costs and productivity loss costs were not included. This is because we assumed that impaired or lost ability to work or participate in leisure activities due to illness would be represented in the disutility of QALY, as per the recommendation of Health Interventions and Technologies for Thai Society (HITAP) (17). Thus, the costs determined in our study comprised direct medical costs and direct non-medical costs. Direct medical costs consisted of hospitalization costs, laboratory investigations, radiological investigations, drug administration, surgery, procedures, referrals, pre-transplantation visits, post-transplantation clinic sessions, admission costs related to KT, cost of dialysis after kidney graft failure, cost of early death due to KT, and hospital services.

We extracted cost data from an electronic medical database of Siriraj Hospital. The data collected were from 140 patients who visited the hospital between January 2009 and December 2019. The costs were obtained by transforming the charges of hospitalizations and outpatient visits of the patients in the cohort using the cost-to-charge ratio of Siriraj Hospital. Our study analyzed and applied costs of 1-year post-KT and subsequent-years post-KT, separately, in the model as we concerned the cost changes in different years post-KT. Annual costs of subsequent-year post-KT were averaged form direct medical costs incurred during 10-year follow-up period. Since there were limited data, we did not include the procurement costs of non-preemptive DDKT, such as donor operations, donor postoperative care, and transportation costs of donor kidney grafts. Direct non-medical costs, consisting of food and transportation, were obtained from the standard unit cost list of a previous study in Thailand (27). All costs were converted to 2020 values using the consumer price index (28) and are reported in 2020 US dollars (1 USD = 31.3 Thai Baht) (29).

### Statistical Analysis

Demographic data and clinical outcomes were analyzed with PASW Statistics for Windows, version 18.0 (SPSS Inc., Chicago, IL, United States). Continuous and categorical variables are presented as the mean ± standard deviation (SD) and frequency (percentage), respectively. The intergroup comparison was analyzed with ANOVA. Estimation of 10-year death-censored graft failure and 10-year survival rates were analyzed by the Kaplan–Meier method and log-rank test. A statistically significant difference was deemed a probability (*P*) value of less than 0.05.

## Results

### Baseline Characteristics

Of 140 patients, 40 had preemptive LRKT, 50 had non-preemptive LRKT, and 50 had non-preemptive DDKT. The mean age of each group was 42.9 ± 15 years, 41.9 ± 15.4 years, and 46.4 ± 9.6 years, respectively (*P* = 0.161). Eighty (57%) patients were men. Although the cause of ESKD for most patients was unknown (46.4%), biopsy-proven IgA nephropathy was the most common known cause (15.1%). The mean waiting times for KT were 1.4 years for preemptive LRKT, 2.3 years for non-preemptive LRKT, and 5.0 years for non-preemptive DDKT (*P* = 0.332). A moderate immunological risk was reported in 87.1% of the patients. The mismatches of human leukocyte antigen and the immunological risks of the 3 groups did not differ. Patient demographic data are presented in Table 2. The 10-year overall patient survival rates were 100.0% for preemptive LRKT, 88.0% for non-preemptive LRKT, and 93% for non-preemptive DDKT. There were no differences in the survival status of the three groups (*P* = 0.257). The 10-year death-censored graft survival rate of each group was not different (preemptive LRKT, 100%; non-preemptive LRKT, 68%; non-preemptive DDKT, 89%; *P* = 0.105). Kaplan-Meier curve of the patient and grafts’ survival rate are shown in Appendix 2, Supplementary Figures 1, 2.

**TABLE 2:** Characteristic of participants.

Characteristics	Preemptive LRKT	Non-preemptive LRKT	Non-preemptive DDKT	*P*-value
*N*	40	50	50	
**Gender; n (%)** Male Female	20 (50) 20 (50)	30 (60) 20 (40)	30 (60) 20 (40)	0.561
Mean age at transplant (yr)	42.9 ± 15.0	41.9 ± 15.4	46.4 ± 9.6	0.161
**Cause of ESKD; n (%)** Chronic glomerulonephritis Diabetic nephropathy IgA nephropathy Lupus nephritis Renal stone Congenital disease FSGS ADPKD Unknown	4 (10) 6 (15) 6 (15) 1 (2.5) 1 (2.5) 2 (5) 2 (5) 6 (15) 12 (30)	7 (14) 7 (14) 9 (18) 1 (2) 1 (2) 0 (0) 1 (2) 0 (0) 23 (48)	3 (6) 6 (12) 7 (14) 2 (4) 0 (0) 1 (2) 0 (0) 1 (2) 30 (60)	0.220
**Health insurance** CSMBS SSS UCS	19 (47.5) 7 (17.5) 14 (35)	8 (16) 20 (40) 22 (44)	18 (36) 21 (42) 11 (22)	0.004
**HLA mismatch; n (%)** HLA MHC Class I MM > 0 HLA MHC Class II MM > 0	35 (87.5) 33 (82.5)	42 (84) 41 (82)	44 (88) 34 (68)	0.820 0.158
**Immunological risk; n (%)** Low Moderate High Very high	2 (5) 37 (92.5) 1 (2.5) 0 (0)	3 (6) 43 (86) 4 (8) 0 (0)	2 (4) 42 (84) 6 (12) 0 (0)	0.566
**Rejection; n (%)** ABMR ACR No rejection	1 (2.5) 3 (7.5) 36 (90)	8 (16) 1 (2) 41 (82)	3 (6) 1 (2) 46 (92)	0.089

*ABMR, antibody mediated rejection; ACR, acute cellular rejection; ADPKD, autosomal dominant polycystic kidney disease; CSMBS, Civil Servant Medical Benefit Scheme; DDKT, deceased-donor kidney transplantation; ESKD, end-stage kidney disease; FSGS, focal segmental glomerulosclerosis; HLA, human leukocyte antigen; LRKT, living-related kidney transplantation; SSS, Social Security Scheme; UCS; Universal Coverage Scheme.*

**FIGURE 2:**
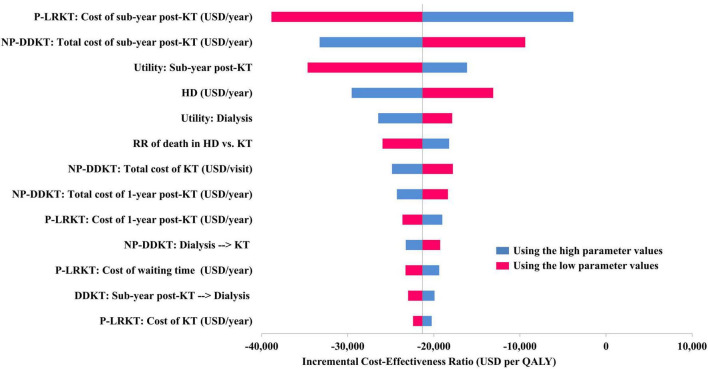
Tornado diagram of preemptive LRKT versus non-preemptive DDKT. HD, hemodialysis; KT, kidney transplantation; NP-DDKT, non-preemptive deceased-donor kidney transplantation; P-LRKT, preemptive living-related kidney transplantation; RR, relative risk.

### Cost-Utility Analysis

Total lifetime costs were $170,356 for preemptive LRKT patients, $187,334 for non-preemptive LRKT patients, and $180,339 for non-preemptive DDKT patients. These costs included pre-transplantation investigations until death. We founded that the direct medical cost of subsequent-year post-KT in IPD setting was higher in non-preemptive LRKT compare with non-preemptive DDKT (6,670 USD/year versus 5,441 USD/year, respectively), as shown in Appendix 2, Supplementary Figure 3. However, the OPD costs and direct non-medical costs were similar in both non-preemptive KT groups. The estimated costs per life year gained were $10,647 for preemptive LRKT, $11,708 for non-preemptive LRKT, and $11,486 for non-preemptive DDKT (Table 3). The incremental costs of the preemptive strategy compared with the other two options were $9,983 for non-preemptive DDKT and $16,977 for non-preemptive LRKT. The QALY gain of the preemptive and non-preemptive LRKT was 0.47 compared with non-preemptive DDKT. Preemptive LRKT was cost-saving compared with DDKT, and it provided more QALY gain using lower total lifetime costs. The scenario analysis comparing non-preemptive LRKT to DDKT showed that non-preemptive LRKT was not cost-effective as the ICERs were $14,944/QALY gained, above the Thai WTP threshold (Table 4). The scenario analysis comparing preemptive LRKT with non-preemptive LRKT indicated that preemptive LRKT was cost-saving because it provided the same level of effectiveness but for lower treatment costs (Table 5).

**TABLE 3:** Costs, outcomes, and cost per life year gained of base-case analysis.

	Discounted costs and outcome 3%			
	Total cost	Life expectancy (yr)	QALY	Cost per LY gained
Preemptive LRKT	170,355.79	16.00	14.12	10,647.23
Non-preemptive DDKT	180,338.83	15.70	13.65	11,486.54
Non-preemptive LRKT	187,333.70	16.00	14.12	11,708.35

*DDKT, deceased-donor kidney transplantation; LRKT, living-related kidney transplantation; KT, kidney transplantation; LY, life-year; QALY, quality-adjusted life years.*

**TABLE 4:** Dominance status of preemptive-LRKT compared with the next most effective modality.

	Total cost	QALY	Incremental Costs	QALYs gained	ICER	Interpretation
Non-preemptive DDKT	180,338.83	13.65	–	–	–	–
Non-preemptive LRKT	187,333.70	14.12	16,977.91	0.47	14,944.42	Dominated*
Preemptive LRKT	170,355.79	14.12	9983.04	0.47 **	–	Cost-saving***

*Dominated by preemptive LRKT. **Compared with non-preemptive DDKT. ***Compared with non-preemptive DDKT and non-preemptive LRKT. DDKT, deceased-donor kidney transplantation; ICER, incremental cost-effectiveness ratio; LRKT, living-related kidney transplantation; QALY, quality-adjusted life years.

**TABLE 5:** Results of scenario-analysis.

	Total cost	QALY	Incremental costs	QALYs gained	ICER
Non-preemptive DDKT	180,338.83	13.65			
Non-preemptive LRKT	187,333.70	14.12	6994.87	0.47	14,944.42

*DDKT, deceased-donor kidney transplantation; LRKT, living-related kidney transplantation; ICER, incremental cost-effectiveness ratio; QALYs, quality-adjusted life years.*

**FIGURE 3:**
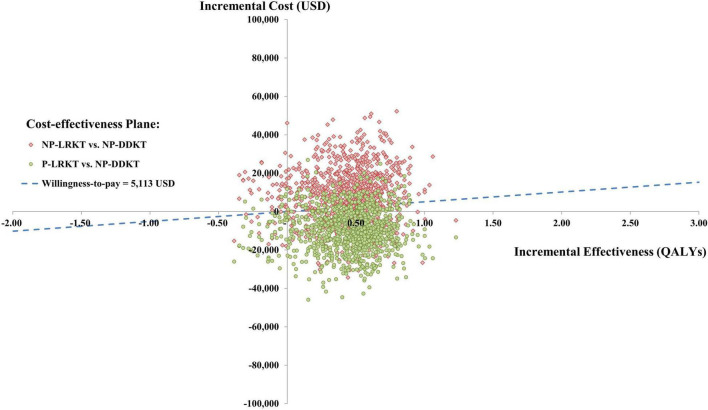
Cost-effectiveness plane of preemptive LRKT and non-preemptive LRKT versus non-preemptive DDKT. NP-DDKT, non-preemptive deceased-donor kidney transplantation; NP-LRKT, non-preemptive living-related kidney transplantation; P-LRKT, preemptive living-related kidney transplantation.

**FIGURE 4:**
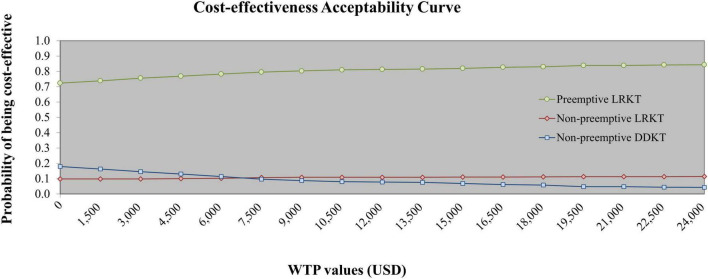
Cost-effectiveness acceptability curve of preemptive LRKT, non-preemptive LRKT, and non-preemptive DDKT. DDKT, deceased-donor kidney transplantation; LRKT, living-related kidney transplantation.

The one-way sensitivity analysis of preemptive LRKT versus non-preemptive DDKT is illustrated in a Tornado diagram (Figure 2). Three most influential parameters were cost of subsequent-year post-LRKT, total cost of subsequent-year post-DDKT, and utility of subsequent-year post-KT, respectively. The sensitivity analyses demonstrated robustness of the model as LRKT remained cost-saving within the varied ranges of all input parameters. The probabilistic sensitivity analysis is depicted as a cost-effectiveness plane (Figure 3). Most of the plots (86.2%) of incremental cost versus incremental QALY of preemptive LRKT versus non-preemptive DDKT were below the WTP threshold line. Of these, 79.4% was considered cost-saving (the plots were in the right lower quadrant of the plane). However, in the scenario analysis (non-preemptive LRKT versus non-preemptive DDKT), 65.7% of the plots were above the WTP threshold line. This meant there was a 65.7% probability that non-preemptive LRKT was not cost-effective compared with non-preemptive DDKT. The probabilities of being cost-effective of the three modalities varied by WTP threshold. They are displayed as cost-effectiveness acceptability curves (Figure 4). Preemptive LRKT remained cost-saving and was the best-buy option.

## Discussion

Kidney transplantation surgery has been performed in Thailand since 1975. However, the use of the procedure was low for several decades in the absence of a policy that the national public health insurance schemes would cover its expenses. Since 2008, the three government health insurance schemes have provided support for the expenses related to all modalities of KT (1). There is evidence that the kidney graft survival and the survival of patients who underwent KT in Thailand have steadily improved over the past decade (6). The preemptive KT strategy in Thailand is only performed as LRKT. Nevertheless, there is no report specifically on preemptive LRKT, including the number of preemptive LRKT cases and the economic evaluation of KT programs relative to preemptive and non-preemptive KT strategies.

Our findings from the model showed that the cost per life year gained with preemptive LRKT was the lowest of the three strategies. The strategy was considered cost-saving as it provided better outcomes in terms of QALY gained and survival rate for lower costs. The sensitivity analyses showed that the result was robust. The costs of non-preemptive DDKT related to the medical and non-medical costs, including costs of procedure and investigation during the pre-KT period to admission, were remarkably higher than preemptive LRKT. We hypothesize that the shorter stay during hospitalization and the fewer complications of the preemptive LRKT option might reduce costs.

Unfortunately, we found that non-preemptive LRKT demonstrated a higher total cost per lifetime than DDKT groups. The explanation was due to the direct medical cost subsequent-year post-KT in IPD setting that was higher in non-preemptive LRKT compare with non-preemptive DDKT. We extracted this data and founded that the patients in non-preemptive LRKT group required more treatment due to the higher incidence of refractory antibody-mediated rejection that required multiple biopsies and numerous courses of treatment. Management of the rejection involved investigations, immunosuppressive drugs, and plasmapheresis caused non-preemptive LRKT to be more costly. A comparison of the ICERs for non-preemptive DDKT and non-preemptive LRKT showed that non-preemptive LRKT was not cost-effective in the current context of Thailand, this result was interpreted based on present WTP threshold of Thailand (5,113 USD/QALY gained). Nevertheless, according to CEAC (Figure 4) which represented the results of PSA, at the WTP threshold of USD 7,500/QALY gained, the probability of non-preemptive LRKT being cost-effective was higher than non-preemptive DDKT (10.7 vs. 9.7%, respectively). Our economic evaluation indicated that preemptive LRKT tended to be the best buying strategy for the management of ESKD patients. However, in the policymaking process, many factors are considered. For example, if a center cannot perform preemptive LRKT, non-preemptive DDKT and LRKT could be alternatives. The capabilities of human resources and KT centers also need to be reviewed. To reduce the costs of management of non-preemptive LRKT, we suggest using a screening and monitoring method to prevent (or at least reduce) the severity of rejection in non-preemptive LRKTs. In general, if the rejection rate of non-preemptive LRKT does not exceed that of non-preemptive DDKT, the non-preemptive LRKT option would be cost-effective.

Our analysis did not use the utility data from our cohort of patients with ESKD due to the absence of quality-of-life data. Instead, we drew upon publications that collected data from Southeast Asian patients to calculate utilities representing the Thai context. Our baseline utility for dialysis patients in Thailand was relatively low (0.68 ± 0.1) (23). This was similar to what had been reported in a study from the Philippines (16). In contrast, a study from Malaysia showed a higher baseline utility (the utility was 0.91 for patients with ESKD) (24). The utility of patients after KT was even greater, ranging between 0.91 and 0.99 (16, 24). These results reflect the improved quality of life after KT in real-world practice. However, no study has specifically investigated the utility of patients who underwent a preemptive strategy. We hypothesize that different health states would occur in patients who receive different ESKD management strategies (such as those who undergo dialysis versus those who do not) and those who receive different KT strategies. Therefore, the utility data we used were different for each health state.

We also determined the 10-year overall patient survival rate and the 10-year graft survival rate for each of the three modalities. The preemptive LRKT strategy showed 100% for both rates. Additionally, preemptive LRKT encountered fewer complications. No patient experienced a severe rejection causing graft failure in the preemptive LRKT group. Furthermore, none of the patients required dialysis after preemptive LRKT. The potential benefits of preemptive LRKT clinical outcomes include fewer complications, avoidance of the dialysis access procedure and its complications, and lower dialysis costs. Our long-term outcomes were similar to those of another study (10, 11).

Several cost-effectiveness studies have been published (12, 13, 16, 24), but data on the financing of preemptive LRKT are lacking. The present work is the first economic evaluation to comprehensively compare the costs of preemptive LRKT and its outcomes in adults with those of other KT strategies in Thai and Southeast Asian settings. The findings of the current work are highly accurate and relevant to the national context for several reasons. Firstly, the economic evaluation process in our study and its results were performed and verified by nephrologists from the Thai Transplantation Society. Secondly, we used local data as much as possible. Since national KT costs were not available, costs were obtained from the transplantation center of the largest university hospital in Thailand. Specialists compiled the data by reviewing medical records, and the charges for related treatments were sourced from the hospital’s electronic database by financial unit personnel. The outputs of the model were expressed as the total lifetime costs of patients undergoing KT. Because we used local costs, the output data should facilitate the development of policies and financial planning for Thailand’s public health insurance schemes.

Other investigations reported that patients with ESKD had higher mortality rates than the general population (20). Mortality hazard ratios of patients with ESKD were used to adjust the death rates of virtual cohorts in these studies. By comparison, we incorporated the latest data for Thailand’s age-specific mortality rates into our model to reflect the life expectancy of Thais. Additionally, our model used input parameters drawn from the most current and applicable studies. We also identified relevant utilities from previous studies and integrated them with local data. Lastly, our study was the first to report long-term consequences (10 years) for Thai KT recipients. The data supported the benefits of the KT procedure.

Our study had several limitations. Firstly, the cost and survival data were taken from a single-center study with a limited sample size. Estimating lifetime costs and outcomes from only 1 cohort might not be adequate to represent the national economic status and long-term clinical outcomes. More sample size is needed to conduct survival analysis in our setting in the future. Nevertheless, the study center has the third-highest KT rate in Thailand (6). Although the patients’ characteristics were consistent with the national data of the Thai Renal Replacement Therapy Registry (6), local cost data may not represent the general cost data for KT for Thailand. This is because Siriraj Hospital is a university hospital, so some costs might be higher than the average for the whole country. It is important to note that our model did not include the DDKT procurement costs. Therefore, the cost of the DDKT group might be underestimated. Cheng et al. (30) reported the estimated organ procurement costs from DDKT using cost function model to demonstrated cost projection. They found that procurement cost of kidney from single-organ transplantation resulted 55,000 USD per kidney. Hence, if the accurate cost data regarding organ procurement is available, we suggest to include them in the future studies Even if procurement costs were included, the interpretation of our study would not have changed: preemptive LRKT was already a cost-saving strategy compared with DDKT. However, the outcomes of non-preemptive LRKT versus non-preemptive DDKT could not be concluded. Next, we did not extract specific cost of subsequent-year post-KT. We decided to use averaged annual costs of subsequent-year post-KT in order to reserve model’s simplicity and minimize error during the process of model development. Another limitation was that we assumed that the maximum age when KT could be performed was 65 years. This age is consistent with the current practice at our center. Nonetheless, the results might not reflect real-world circumstances if the KT guidelines change. Furthermore, we did not compare preemptive LRKT with other dialysis modalities since doing so was outside the scope of the study. However, a study in China indicated that KT is cost-effective relative to hemodialysis and peritoneal dialysis (31). Moreover, if the results of this study are intended to be used elsewhere, they should be interpreted with caution due to the different socioeconomic statuses and public-sector health insurance policies for KT. Nevertheless, our methodology and findings should be helpful examples for low- and middle-income countries.

This study supports the goal of promoting preemptive LRKT in Thailand. However, the challenges of policy improvement associated with patient education, late referral, and a lower rate of preemptive LRKT should be fully considered. Improving the rate of effective preemptive LRKT is a primary objective. Previous studies demonstrated that more than half of patients with ESKD did not anticipate ever undergoing KT and needed to be encouraged to undergo preemptive LRKT because they had difficulty understanding the benefits of the procedure (32). To promote the acceptance and uptake of this treatment option, it is essential to focus on the education of patients and healthcare providers.

In Thailand, preemptive LRKT was cost-saving compared with non-preemptive KT strategies. Our findings should be considered part of evidence-based policy development to promote a preemptive LRKT strategy among patients with ESKD in Thailand. We suggest further studies that compare the cost-utility of preemptive LRKT and dialysis modalities. Doing so will ensure that preemptive LRKT is appropriate as the first choice for managing patients with ESKD.

## Data Availability Statement

The raw data supporting the conclusions of this article will be made available by the authors, without undue reservation.

## Ethics Statement

The studies involving human participants were reviewed and approved by the Siriraj Institutional Review Board (approval number: 946/2020). Written informed consent for participation was not required for this study in accordance with the national legislation and the institutional requirements.

## Author Contributions

AP, PP, CK, and AV designed and conceptualized the study, and revised the manuscript. AP collected the data and drafted the manuscript. AP, CK, and PP performed the data analyses and interpretations. All authors contributed to the article and approved the publication of the submitted version.

## Conflict of Interest

The authors declare that the research was conducted in the absence of any commercial or financial relationships that could be construed as a potential conflict of interest.

## Publisher’s Note

All claims expressed in this article are solely those of the authors and do not necessarily represent those of their affiliated organizations, or those of the publisher, the editors and the reviewers. Any product that may be evaluated in this article, or claim that may be made by its manufacturer, is not guaranteed or endorsed by the publisher.

## References

[1] Kanjanabuch T Takkavatakarn K. Global dialysis perspective: Thailand. *Kidney360.* (2020) 1:671–5. 10.34067/KID.0000762020 35372930 PMC8815550

[2] Thai Transplantation Society. *Annual Report Thailand Renal Replacement Therapy 2007-2019 (Th).* (2021). Available online at: https://www.nephrothai.org/annual-report-thailand-renal-replacement-therapy-2007-2019-th/ (accessed March 7, 2021).

[3] Schnuelle P Lorenz D Trede M Van Der Woude FJ. Impact of renal cadaveric transplantation on survival in end-stage renal failure: evidence for reduced mortality risk compared with hemodialysis during long-term follow-up. *J Am Soc Nephrol JASN.* (1998) 9:2135–41. 10.1681/ASN.V9112135 9808102

[4] Wolfe RA Ashby VB Milford EL Ojo AO Ettenger RE Agodoa LYC Comparison of mortality in all patients on dialysis, patients on dialysis awaiting transplantation, and recipients of a first cadaveric transplant. *N Engl J Med.* (1999) 341:1725–30. 10.1056/NEJM199912023412303 10580071

[5] Friedewald JJ Reese PP. The kidney-first initiative: what is the current status of preemptive transplantation? *Adv Chronic Kidney Dis.* (2012) 19:252–6. 10.1053/j.ackd.2012.05.001 22732045 PMC3384698

[6] Thai Transplantation Society. (2021). Available online at: http://www.transplantthai.org/data/annual_report/1/2020%20TH.pdf (accessed March 7, 2021).

[7] Vats AN Donaldson L Fine RN Chavers BM. Pretransplant dialysis status and outcome of renal transplantation in North American children: a NAPRTCS Study. North American pediatric renal transplant cooperative study. *Transplantation.* (2000) 69:1414–9. 10.1097/00007890-200004150-00035 10798764

[8] Mange KC Joffe MM Feldman HI. Effect of the use or nonuse of long-term dialysis on the subsequent survival of renal transplants from living donors. *N Engl J Med.* (2001) 344:726–31. 10.1056/NEJM200103083441004 11236776

[9] Dean P Heien H Swanson K Borah B Sangaralingham L Naessens J *The Cost Savings of Preemptive Kidney Transplantation.* (2022). Available online at: https://atcmeetingabstracts.com/abstract/the-cost-savings-of-preemptive-kidney-transplantation/ (accessed February 7, 2022).

[10] Jay CL Dean PG Helmick RA Stegall MD. Reassessing preemptive kidney transplantation in the united states: are we making progress? *Transplantation.* (2016) 100:1120–7. 10.1097/TP.0000000000000944 26479285 PMC4989865

[11] Amaral S Sayed BA Kutner N Patzer RE. Preemptive kidney transplantation is associated with survival benefits among pediatric patients with end-stage renal disease. *Kidney Int.* (2016) 90:1100–8. 10.1016/j.kint.2016.07.028 27653837 PMC5072842

[12] Ludbrook A. A cost-effectiveness analysis of the treatment of chronic renal failure. *Appl Econ.* (1981) 13:337–50. 10.1080/00036848100000004

[13] Klarman HE Francis JO Rosenthal GD. Cost effectiveness analysis applied to the treatment of chronic renal disease. *Med Care.* (1968) 6:48–54. 10.1097/00005650-196801000-00005

[14] Garner TI Dardis R. Cost-effectiveness analysis of end-stage renal disease treatments. *Med Care.* (1987) 25:25–34. 10.1097/00005650-198701000-00004 3100878

[15] Aswapokee N Vaithayapichet S Heller RF. Pattern of antibiotic use in medical wards of a university hospital, Bangkok, Thailand. *Rev Infect Dis.* (1990) 12:136–41. 10.1093/clinids/12.1.136 2300735

[16] Bayani DBS Almirol BJQ Uy GDC Taneo MJS Danguilan RS Arakama M-HI Filtering for the best policy: an economic evaluation of policy options for kidney replacement coverage in the Philippines. *Nephrology.* (2021) 26:170–7. 10.1111/nep.13830 33207027

[17] Chaikledkaew U Kittrongsiri K. Guidelines for health technology assessment in Thailand (second edition)-the development process. *J Med Assoc Thai.* (2014) 97:4. 24964693

[18] Briggs A Sculpher M Claxton K. *Decision Modelling for Health Economic Evaluation.* Oxford: OUP (2006).

[19] GHO. *Life Tables by Country – Thailand.* Geneva: World Health Organization (2021).

[20] Choi H Kim M Kim H Lee JP Lee J Park JT Excess mortality among patients on dialysis: comparison with the general population in Korea. *Kidney Res Clin Pract.* (2014) 33:89–94. 10.1016/j.krcp.2014.04.001 26877956 PMC4714183

[21] Ingsathit A Kamanamool N Thakkinstian A Sumethkul V. Survival advantage of kidney transplantation over dialysis in patients with hepatitis C. *Transplantation.* (2013) 95:943–8. 10.1097/TP.0b013e3182848de2 23425817

[22] Ying T Shi B Kelly PJ Pilmore H Clayton PA Chadban SJ. Death after kidney transplantation: an analysis by era and time post-transplant. *J Am Soc Nephrol.* (2020) 31:2887. 10.1681/ASN.2020050566 32908001 PMC7790214

[23] Teerawattananon Y Mugford M Tangcharoensathien V. Economic evaluation of palliative management versus peritoneal dialysis and hemodialysis for end-stage renal disease: evidence for coverage decisions in Thailand. *Value Health.* (2007) 10:61–72. 10.1111/j.1524-4733.2006.00145.x 17261117

[24] Bavanandan S Yap Y-C Ahmad G Wong H-S Azmi S Goh A. The cost and utility of renal transplantation in Malaysia. *Transplant Direct.* (2015) 1:e45. 10.1097/TXD.0000000000000553 PMC4946449 27500211

[25] EuroQol Group. EuroQOL—a new facility for the measurement of health-related quality of life. *Health Policy.* (1990) 16:199–208. 10109801 10.1016/0168-8510(90)90421-9

[26] Herdman M Gudex C Lloyd A Janssen M Kind P Parkin D Development and preliminary testing of the new five-level version of EQ-5D (EQ-5D-5L). *Qual Life Res.* (2011) 20:1727–36. 10.1007/s11136-011-9903-x 21479777 PMC3220807

[27] Riewpaiboon A. Measurement of costs. *J Med Assoc Thail.* (2008) 91 Suppl 2:S28–37. 19253485

[28] Consumer Price Index of Thailand. *Economic and Trade Indices Database (ETID).* New Delhi: Bureau of Trade and Economic Indices, Ministry of Commerce (2020).

[29] Bank of Thailand. *Historical Foreign Exchange Rates.* Phra Nakhon: Bank of Thailand (2020).

[30] Cheng XS Held PJ Dor A Bragg-Gresham JL Tan JC Scandling JD The organ procurement costs of expanding deceased donor organ acceptance criteria: evidence from a cost function model. *Am J Transplant.* (2021) 21:3694–703. 10.1111/ajt.16617 33884757

[31] Wu H Li Q Cai Y Zhang J Cui W Zhou Z. Economic burden and cost-utility analysis of three renal replacement therapies in ESRD patients from Yunnan Province, China. *Int Urol Nephrol.* (2020) 52:573–9. 10.1007/s11255-020-02394-1 32009220

[32] Weng FL Mange KC. A comparison of persons who present for preemptive and nonpreemptive kidney transplantation. *Am J Kidney Dis.* (2003) 42:1050–7. 10.1016/j.ajkd.2003.07.007 14582049

